# Ozonated Water Modulates Cell Proliferation and Vascular Density in Solid Ehrlich Tumor of Mice

**DOI:** 10.3390/cancers18050733

**Published:** 2026-02-25

**Authors:** Diego Pereira de Araújo, Eduardo de Paula Nascente, Juliana Santana de Curcio, Mariana Batista Rodrigues Faleiro, Emmanuel Arnhold, Elisângela de Paula Silveira Lacerda, Moema Pacheco Chediak Matos, Carlos Eduardo Fonseca-Alves, Veridiana Maria Brianezi Dignani de Moura

**Affiliations:** 1 School of Veterinary and Animal Science, Federal University of Goiás, Goiânia 74690-900, Goiás, Brazil; diegoaraujomv@outlook.com (D.P.d.A.); eduardopaula@pucgoias.edu.br (E.d.P.N.); marianafavet@ufg.br (M.B.R.F.); emmanuelarnhold@yahoo.com.br (E.A.); mpcmatos@ufg.br (M.P.C.M.); 2 Institute of Biological Sciences, Federal University of Goiás, Goiânia 74001-970, Goiás, Brazil; julianadecurcio1@gmail.com (J.S.d.C.); elacerda@ufg.br (E.d.P.S.L.); 3 Department of Veterinary Surgery and Animal Reproduction, São Paulo State University (UNESP), Botucatu 18618-681, Brazil; carlos.e.alves@unesp.br

**Keywords:** anti-CD31, anti-Ki-67, immunohistochemistry, experimental oncology, ozone therapy

## Abstract

Background: The solid Ehrlich tumor (SET) is a transplantable experimental neoplasm that mimics mammary adenocarcinoma in female mice, widely used to investigate tumor physiology, behavior, and therapeutic interventions. Among emerging approaches, ozone therapy has gained attention in human and veterinary medicine, prompting studies to clarify its mechanisms and potential applications. This study evaluated vascular and tumor cell proliferation in SET of mice treated with ozonated water under different protocols. Methods: A total of 99 animals were allocated into four groups: ozonated water at 104 mM/5 ppm (G1, *n* = 30), 208 mM/8 ppm (G2, *n* = 30), vehicle control with 0.9% saline (G3, *n* = 30), and negative control (GCN, *n* = 9). Subgroups were established according to administration routes (intratumoral or peritumoral), number of applications (one or two), and observation periods (24 h, five days, or 30 days). Immunohistochemistry with anti-CD31 and anti-Ki-67 antibodies assessed vascular and cellular proliferation, respectively, considering peri- and intratumoral regions. Results: Increased CD31 expression was detected at 30 days in treated groups compared to controls, particularly after two intratumoral applications and in all peritumoral (PT) protocols. Ki-67 expression was reduced after five days in the treated groups, indicating decreased cell proliferation relative to controls. A positive correlation was observed between peri- and intratumoral CD31 immunostaining. Conclusion: Ozonated water reduced tumor cell proliferation in the medium term, but treatment discontinuation favored increased vascular density in the long term. These findings suggest caution in the oncological use of ozone, as it may present both antineoplastic and tumor-promoting effects depending on treatment conditions.

## Simple Summary

The solid Ehrlich tumor (SET) is an experimental model in mice used to study cancer. Ozone therapy has been suggested as a complementary treatment in medicine, but its effects on tumors are not fully understood. This study tested whether ozonated water could influence tumor blood vessels and cell growth. Mice with SET were treated with different doses of ozonated water, given either inside or around the tumor, and compared to control groups. The researchers measured blood vessel formation by CD31 and tumor cell growth by Ki-67. Results showed that ozonated water reduced tumor cell proliferation after five days, and at 30 days, it increased blood vessel density, especially with repeated applications. These findings suggest that ozone therapy may have both beneficial and harmful effects on tumors, helping to slow cell growth in the short term, but possibly favoring tumor progression over time.

## 1. Introduction

Ehrlich tumor (ET) is a transplantable neoplasm, corresponding to mammary adenocarcinoma in female mice, and is widely used in experimental oncological evaluations, particularly those related to tumor physiology, behavior, and therapy [1]. It is employed in preclinical experimental models for mammary neoplasms [2,3,4], especially in its solid form (solid Ehrlich tumor—SET) [5].

In oncogenesis, angiogenesis and the factors influencing its development are closely associated with tumor progression, which also occurs in SET [6].Concomitant with tumor growth, this process develops through two main pathways: one triggered by endothelial growth factors released by neoplastic cells (germinal growth) and the other induced by stimulation of pre-existing vessels (co-option) [6,7].

SET exhibits the expression of several angiogenesis markers, such as VEGF, VEGFR2, and CD31, the latter of which may show a positive correlation with the reduction in cell proliferation as expressed by Ki67 [8,9,10,11], similar to what has been reported in melanoma [12] and gastrointestinal stromal tumor [13].

The cell proliferation rate in different tumors is commonly assessed by immunomarkers, with Ki67 being the most widely used. The proportion of Ki67 immunostaining in tumor tissues represents a prognostic parameter [14,15]. This marker is mainly expressed in the S phase of the cell cycle, although it has also been detected in other phases, such as interphase and mitosis [16]. Consequently, high Ki67 indices are associated with poor prognosis, including recurrence and reduced survival rates [17].

Ozone therapy has been extensively studied in both human and veterinary medicine due to its low cost, safety, and minimal invasiveness [18]. However, further investigation is required to elucidate the mechanisms of action of ozone (O_3_) in antitumor therapy, particularly in solid tumors, and to assess its effects on microvascular density and cell proliferation rates.

Among the different forms of treatment, ozonated water has shown advantages over other forms of application, such as autohemotherapy and gas insufflation, in several cancer treatments [19]. Compared to other modalities, ozonated water shows greater solubility, a lower risk of inhalation or loss of the active compound, ease of handling during therapy, greater antimicrobial efficacy, and indirect biological effects mediated by reactive oxygen species (ROS) [20,21,22]. Given this, the aim of the present study was to evaluate the proliferation of blood vessels and tumor cells in SET experimentally induced in mice and subjected to treatment with ozonized water.

## 2. Materials and Methods

This study was approved by the Ethics Committee on Animal Use (CEUA), protocol no. 058/19, of the Federal University of Goiás (UFG). The experimental procedures were conducted at the Animal Facility of the Laboratory of Radiobiology and Mutagenesis, Institute of Biological Sciences I (ICB I), and at the Laboratory of Pathology, School of Dentistry, UFG.

A total of 102 mice (*Mus musculus*), Swiss strain, albino, female, eight weeks old, weighing approximately 40 g, were obtained from the Production and Science Center for Biomodels (CPCBio) of UFG. Initially, the ascitic form of ET was induced in three mice (P1). Subsequently, ascitic ET cells were harvested, assessed for viability [1], and inoculated into 99 mice (P2) to induce the development of SET. Inoculation consisted of 0.5 mL of saline solution containing 5 × 10^6^ cells/mL, administered subcutaneously into the right dorsolateral region of the mice.

The P2 mice were visually monitored and subjected to low-invasiveness reaction tests for seven days, corresponding to the peak of SET growth, following routine animal welfare monitoring procedures conducted in accordance with standardized guidelines and manuals [1,23]. After this period, and following confirmation of tumor growth, treatment protocols with ozonized water were initiated. For this purpose, four groups and 33 subgroups were established, each comprising six mice. Groups G1 (N = 30) and G2 (N = 30) received ozonized water at concentrations of 5 ppm and 8 ppm, respectively; the vehicle control group (G3, N = 30) received 0.9% saline solution; and the negative control group (GCN, N = 9) received no treatment. In all groups, except for the NCG, two administration routes were tested—peritumoral subcutaneous (SCP) and intratumoral (IT)—as well as single (1×) or double (2×) applications, with a 24 h interval between administrations, adapted from Kuroda et al. [19].

For groups G1, G2, and G3, animals were euthanized at the following time points: (1) M1/G1, M1/G2, and M1/G3—mice receiving a single application of ozonized water or saline and euthanized after 24 h; (2) M2/G1, M2/G2, and M2/G3—mice receiving a single application and euthanized after five days; (3) M3/G1, M3/G2, and M3/G3—mice receiving a single application and euthanized after thirty days; (4) M4/G1, M4/G2, and M4/G3—mice receiving two applications and euthanized five days after the first application; and (5) M5/G1, M5/G2, and M5/G3—mice receiving two applications and euthanized thirty days after the first application (Table 1), according to criteria adapted from Kuroda et al. [19]. The negative control groups (GCN = 09) did not undergo any treatment, allowing the tumor to grow without intervention. These animals were euthanized beginning on the seventh day after the peak of tumor growth, at the following intervals: 24 h, five days, and thirty days post-peak growth.

The ozonated water-treated groups and the vehicle control group received the same administration volume (1 mL) per application, via the intratumoral or peritumoral routes, according to the experimental protocol. For all groups (Table 1), euthanasia was performed to assess the short-term (24 h), medium-term (five days), and long-term (thirty days) effects of ozonized water on SET. The procedure consisted of an overdose of isoflurane at the highest achievable concentration (>5%), via an anesthesia system connected to the PerkinElmer FMT 4000 Fluorescence Tomography System (FMT^®^) (PerkinElmer, São Paulo, Brazil), using a dedicated acrylic chamber for rodents, without image acquisition, followed by cervical dislocation [24].

After euthanasia, the SET was excised from each mouse with a 1 cm lateral margin. Samples were collected, fixed in 10% buffered formalin for 48 h, and subsequently preserved in 70% ethanol until processing and paraffin embedding for subsequent immunohistochemical evaluation.

Three histological sections were prepared from each of the 99 SET samples: one for hematoxylin and eosin (HE) staining, and two for immunohistochemical analysis with anti-Ki67 and anti-CD31 antibodies. In the evaluation of HE-stained sections, it was ensured that the corresponding sections for anti-CD31 and anti-Ki67 immunohistochemistry presented: (1) sufficient tumor growth to allow the reliable counting of at least five areas of vascular proliferation; and (2) tumor regions arranged in sheets, avoiding necrotic areas, as described by Marien et al. [25], Saadeh et al. [26], and Feng et al. [27]. All immunohistochemical evaluations were performed by observers blinded to the experimental groups.

For immunostaining with anti-CD31 and anti-Ki67 antibodies, 4 µm histological sections were mounted on charged slides (ImmunoSlide^®^, Easypath, Indaiatuba, SP, Brazil), dried in an oven at 56 °C for 24 h, deparaffinized, hydrated, and rinsed in distilled water. Antigen retrieval was performed using citrate buffer (pH 6.0) in a pressure cooker (Model S2800, Dako Cytomation, Carpinteria, CA, USA). Sections were immersed in hydrogen peroxide solution to block endogenous peroxidase activity (EP-11-20521, Easy Path, Indaiatuba, São Paulo, Brazil) for 10 min, rinsed in distilled water, and immersed in a protein background blocking solution (EP-12-20531, EasyPath, Indaiatuba, São Paulo, Brazil) for 10 min.

Slides were then incubated for 18 h with the primary antibodies: anti-CD31/Pecam1 Antibody Picoband™ (Boster Biological Technology^®^, rabbit polyclonal, catalog no. A01513-3, Pleasanton, CA, USA) and anti-Ki67/Rabbit Monoclonal Antibody, concentrated and pre-diluted—SP6 (BioCare Medical^®^, rabbit monoclonal, catalog no. 901-325-042921, Pacheco, CA, USA), at dilutions of 1:150 and 1:100, respectively. After two washes with phosphate-buffered saline (PBS) for 5 min, the sections were incubated with a polymer-based secondary antibody, N-Histofine^®^ (Simple Stain Rat MAX PO (MULTI), Nichirei Biosciences Inc., Tokyo, Japan), for 30 min. Slides were rinsed again in PBS, and the reactions were visualized by incubation with diaminobenzidine (DAB) chromogen solution for 5 min, followed by counterstaining with Mayer’s hematoxylin.

SET samples immunostained with anti-CD31 were subjected to microvessel density (MVD) assessment, in which vascular proliferation regions were identified at 20× magnification, followed by quantification of vascular density at 40× in at least five fields, considering both intratumoral and peritumoral regions, adapted from Fox et al. [28]. Areas with necrosis, inflammation, or ulceration were excluded, with preference given to regions containing at least 75% neoplastic tissue [25]. These areas were captured as images using the ToupView^®^ software version 4.1, and vessels with representative staining and visible lumina in cross-sections were marked and subsequently counted.

The quantification of anti-Ki67 immunostaining was performed semi-automatically in ten high-power fields (40×) of SET areas containing more than 75% neoplastic cellularity, captured as images. Cell counting was performed using ImageJ^®^ software version 1.54 to determine the percentage of immunostained cells, according to criteria adapted from Saadeh et al. [26] and Feng et al. [27].

Data were tested for residual normality using the Shapiro–Wilk test. Group comparisons were performed by analysis of variance (ANOVA) followed by the Scott–Knott multiple comparison post-test, being group comparisons only between animals in the same euthanasia time (Table 1). Correlations between variables were also evaluated, and a significance level of 5% was adopted. Statistical analyses were carried out using the R Core Team software version 4.5.2 with the easyanova package [29].

## 3. Results

**Figure 1:**
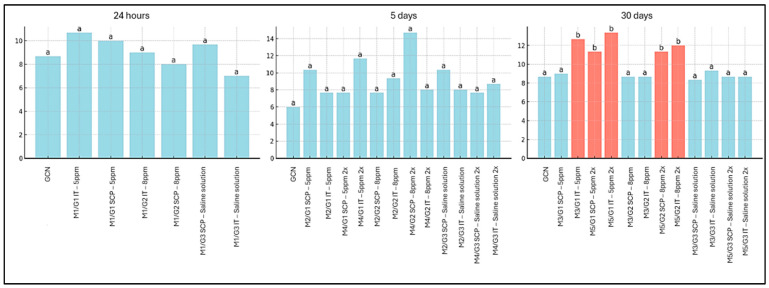
Evaluation of vascular microdensity in the intratumoral region of solid Ehrlich tumor in mice, measured by vessel counting immunostained with the anti-CD31 antibody, considering the 24 h, 5-day, and 30-day time points, as well as different doses and routes of administration of ozonated water. Legend: Different colors and fonts between columns are different from each other (*p* < 0.05). G1: Group treated with ozonated water at a concentration of 5 ppm; G2: Group treated with ozonated water at a concentration of 8 ppm; G3: Vehicle control group treated with 0.9% saline; SCP: subcutaneous peritumoral route of administration; IT: intratumoral route of administration. Groups M1, M3, and M5 correspond to animals euthanized at 24 h, 5 days, and 30 days after the first application, respectively.

**Figure 2:**
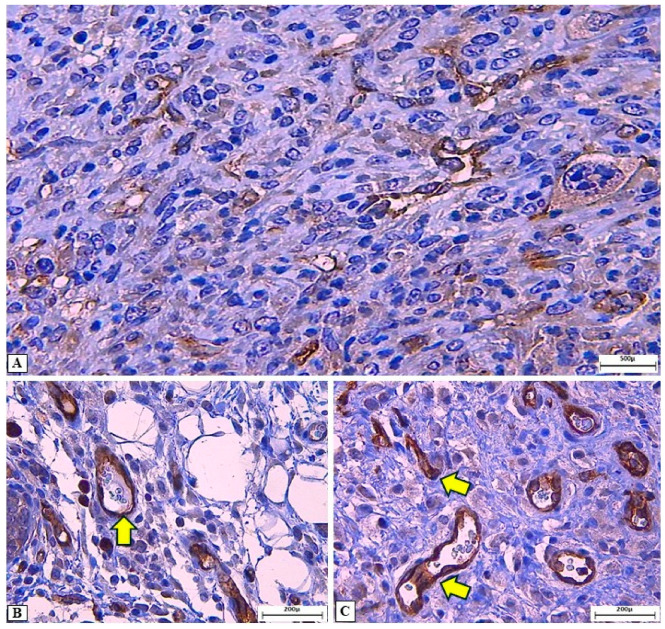
Photomicrographs of solid Ehrlich tumor in mice, treated with different ozonated water protocols and subjected to vascular microdensity assessment with the anti-CD31 antibody. (**A**) Cytoplasmic immunostaining in endothelial cells. (**B**,**C**) Endothelial (arrows) and inflammatory cells immunostaining in the peritumoral and intratumoral regions of SET, respectively. Immunohistochemistry (IHC), anti-CD31; original magnification: 10× (**A**) and 40× (**B**,**C**).

In the assessment of intratumoral vascular microdensity using the anti-CD31 antibody, no differences were observed between treated groups and controls (GCN and G3) at the 24 h and five-day evaluation time points (Figure 1). However, at 30 days post-treatment, an increase in vascular immunostaining was detected in groups M3/G1 IT (5 ppm), M5/G1 IT (5 ppm; two applications), M5/G1 SCP (5 ppm; two applications), M5/G2 SCP (8 ppm; two applications), and M5/G2 IT (8 ppm; two applications), when compared to the GCN, G3, and the other groups treated with ozonated water (Figure 1). Figure 2 illustrates the immunostaining pattern with the anti-CD31 antibody in the intratumoral and peritumoral regions of SET treated with ozonated water. An intense and well-defined cytoplasmic immunostaining was observed in endothelial cells, highlighting the tumor vascular network, which was predominantly more extensive in the peritumoral region. Additionally, the anti-CD31 also revealed mononuclear inflammatory cells distributed in the peritumoral and intratumoral regions of the SET.

**Figure 3:**
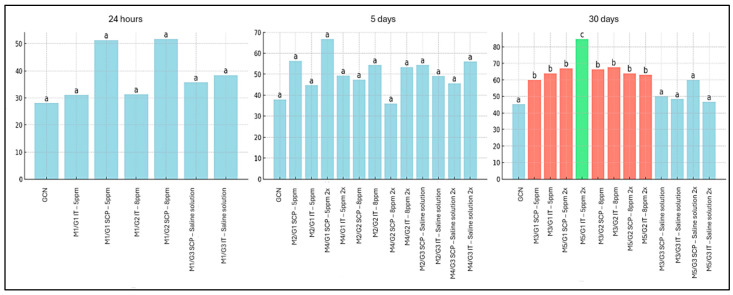
Evaluation of microvascular density in the peritumoral region of the solid Ehrlich tumor in mice, measured by vessel counting, immunostained with the anti-CD31 antibody, considering the evaluation time points of 24 h, 5 days, and 30 days, as well as different doses and routes of administration of ozonated water. Legend: Different colors and fonts between columns are different from each other (*p* < 0.05). G1: Group treated with ozonated water at a concentration of 5 ppm; G2: Group treated with ozonated water at a concentration of 8 ppm; G3: Vehicle control group treated with 0.9% saline; SCP: subcutaneous peritumoral route of administration; IT: intratumoral route of administration. Groups M1, M3, and M5 correspond to animals euthanized at 24 h, 5 days, and 30 days after the first application, respectively.

In the evaluation of vascular microdensity in the peritumoral region of SET treated with ozonated water (Figure 3), no differences were observed between treated groups and negative controls (GCN and G3) at the 24 h and five-day time points. However, in the groups assessed 30 days after treatment, vascularization was increased in all animals treated with O_3_ when compared to GCN and G3. Furthermore, the SETs of the M5/G1 IT group (5 ppm; two applications) exhibited an intratumoral vascular microdensity even greater than that observed in the other groups, whether treated or untreated with ozonated water (Figure 3).

**Figure 4:**
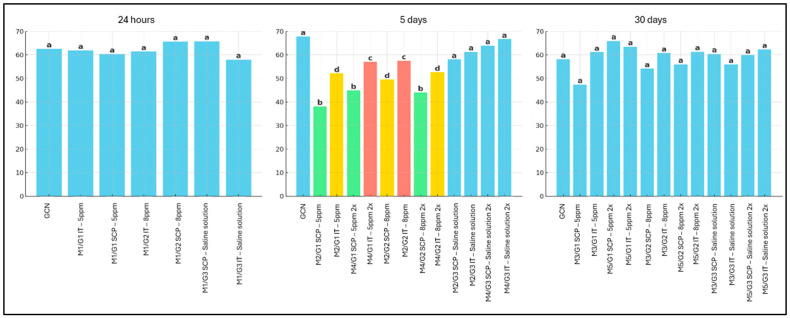
Evaluation of the cell proliferation in the intratumoral region, measured by the percentage of immunolabeled cells in the solid Ehrlich tumor of mice, using the anti-Ki-67 antibody, considering the evaluation time points of 24 h, five days, and 30 days, as well as different doses and routes of administration of ozonated water. Legend: Different colors and fonts between columns are different from each other (*p* < 0.05). G1: Group treated with ozonated water at a concentration of 5 ppm; G2: Group treated with ozonated water at a concentration of 8 ppm; G3: Vehicle control group treated with 0.9% saline; SCP: Subcutaneous Peritumoral route of administration; IT: Intratumoral route of administration.

**Figure 5:**
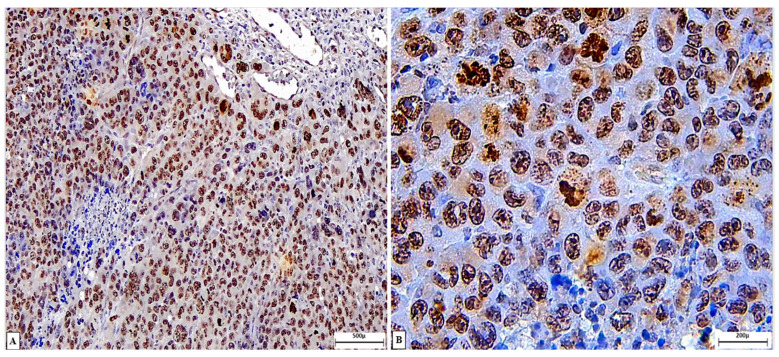
Photomicrographs of a solid Ehrlich tumor of mice in mice subjected to cell proliferation assessment with the anti-Ki67 antibody. (**A**) Intratumoral region overview of immunolabeled tumor cells. IHC, 10× objective; (**B**) detail of nuclear immunostaining. IHC, 40× objective.

The M2/G1 SCP, M4/G1 SCP, and M4/G2 SCP groups exhibited the lowest cell proliferation rates, followed by M2/G1 IT, M2/G2 SCP, and M4/G2 IT, all receiving two applications. The highest proliferative indices were observed in M4/G1 IT (two applications) and M2/G2 IT (one application) (Figure 4). Figure 5 illustrates the immunostaining pattern using the anti-Ki67 antibody in SET treated with ozonated water. An overview of neoplastic cells with positive immunostaining is observed, with intense and predominantly nuclear Ki-67 labeling, indicative of high proliferative activity, occasionally associated with discrete cytoplasmic immunoreactivity in some tumor cells.

In the correlation analysis, a slight increase in vascular microdensity was observed between the intratumoral and peritumoral regions, indicating that vascular expansion in one region was associated with a corresponding increase in the other. On the other hand, no correlation was found between vascular microdensity and cell proliferation in SET treated with ozonated water across different doses, numbers of applications, routes of administration, and evaluation time points (Table 2).

## 4. Discussion

The evaluation of microvascular density (MVD) based on the quantification of anti-CD31 immunolabeling revealed an increase in the O_3_-treated groups, particularly in the long-term assessment (30 days). This finding suggests that the discontinuation of ozonotherapy may favor late-stage vascularization, which contrasts with other studies in which no evidence of neovascularization was observed when approaches such as autohemotherapy with ozonated blood [30] or rectal insufflation [31] were employed. It should be noted that the period of tumor development observation in those studies was shorter than in the present investigation.

The production of reactive oxygen species (ROS) resulting from O_3_ oxidation in blood and tissue fluids may stimulate the release of vascular endothelial growth factor (VEGF) [32,33], which could account for the increased vascularization observed in SET treated with ozonated water. However, the late increase in microvascular density may be explained by two distinct mechanisms. First, the discontinuation of O_3_ therapy would favor the reestablishment of the angiogenic process, since the neovascularization in neoplasms may occur at more advanced stages [33,34,35]. Second, the possibility of a late pro-angiogenic signaling process induced either by ozone itself or by reactive oxygen species (ROS) cannot be excluded. As highlighted by Ogut [36], the late pro-angiogenic effects of ozone therapy, as well as the exact dose–response dynamics and long-term safety of ozone, remain uncertain. According to the author, ozone therapy may promote a more comprehensive and sustained pro-angiogenic effect by stimulating multiple angiogenic mediators, not only through redox signaling mechanisms, but also via immunomodulatory pathways.

In this context, ozonotherapy has been associated with increased expression not only of vascular endothelial growth factor (VEGF), but also of transforming growth factor-beta (TGF-β), and platelet-derived growth factor (PDGF) [37]. This stimulation may be explained by increased local concentrations of hydrogen peroxide (H_2_O_2_), which acts as a secondary messenger capable of activating NF-κB, nitric oxide pathways, and protein kinases, ultimately leading to the release of cytokines such as TNF-α, IL-1, IL-8, IFN-γ, and TGF-β1, which are involved in the vascular remodeling observed following ozone administration [38].

It is widely recognized that increased blood vessel proliferation in neoplasms may be beneficial during the initial phase of tumor growth, as it promotes hyperoxia within the tumor microenvironment, thereby preventing the activation of autophagic mechanisms in neoplastic cells [39]. However, it is argued that the increase in vascularization in SET after 30 days may be associated with an unfavorable prognosis, as previously demonstrated in human mammary carcinomas, in which high anti-CD31 immunolabeling correlates with enhanced primary tumor growth and an increased risk of metastasis, particularly in the long term [40,41,42]. Accordingly, these findings reinforce the notion that discontinuation of ozonated water treatment leads to a late increase in microvascular density, followed by marked SET growth, reflecting the high malignant and proliferative potential of this neoplasm.

It is also well established that chemotherapy may induce genomic instability in tumors, which can favor a more aggressive biological behavior and frequently result in the development of chemoresistance [43,44]. In this context, and with due consideration of the limitations of such comparisons, it is hypothesized that the prolonged absence of ozonated water treatment may, in some manner, have contributed to the resumption of proliferation of neoplastic cells that were not eliminated during therapy. Consequently, this may have stimulated new vessel formation, thereby justifying the observed increase in anti-CD31 immunolabeling and opening avenues for future investigations.

With respect to the evaluation of cellular proliferation through the quantification of anti-Ki67 immunoexpression, a reduction in intratumoral proliferation was observed in SET from the O_3_-treated groups evaluated in the medium term (five days), with the lowest proliferative activity recorded in SET treated with two peritumoral subcutaneous applications. Nevertheless, the cellular proliferation values remained elevated when compared with those reported in human tumors [45], a difference that may be attributed to the SET lineage employed in this study, which exhibits a high proliferative potential.

In agreement with the present findings, other studies have demonstrated that O_3_-based treatments have the potential to reduce Ki-67 immunoexpression, both when used as an adjuvant therapy [46] and as a stand-alone therapeutic approach in cancer treatment [47], as well as in non-neoplastic conditions, such as in the cerebellum of mice treated with intraperitoneal ozonation [48]. In this context, it is postulated that O_3_ may contribute to a reduction in cellular proliferation rates, particularly during the G2 and M phases of the cell cycle, by promoting intracellular ROS accumulation and glutathione depletion, thereby inactivating the expression of key proliferation-related factors involved in neoplastic development, including phosphatidylinositol 3-kinase (PI3K), protein kinase B (PKB), and NF-κB [49]. However, similarly to what was observed for microvascular density, the beneficial effects of O_3_ on cellular proliferation in murine SET do not appear to be sustained in the long term, as evidenced by the resumption of tumor cell proliferation following treatment discontinuation and evaluation at 30 days.

With regard to the antibodies employed in this study for the evaluation of microvascular density and cellular proliferation in murine SET, it is well established that there is a strong correlation between anti-CD31 and anti-Ki67 immunolabeling, indicating greater aggressiveness in mammary tumors [50]. However, although microvascular density at 30 days increased in most of the O_3_-treated groups compared with untreated controls, the resumption of tumor cell proliferation did not occur to the same extent.

The absence of a significant correlation between microvascular density (CD31) and tumor cell proliferation (Ki-67) represents a relevant finding of this study. Although angiogenesis is commonly regarded as a facilitator of tumor growth [35,40], vascular expansion and cellular proliferation are not necessarily synchronized processes in all contexts. This dissociation reinforces the concept that angiogenic remodeling may occur independently of proliferative activity, particularly in dynamic experimental models involving redox-modulating agents, such as ozonated water. These findings indicate that modulation of CD31 expression should not be interpreted as a direct indicator of tumor growth in all cases, highlighting the complexity of tumor–microenvironment interactions and the need for cautious interpretation of angiogenic markers when evaluated in isolation, without additional associated parameters.

Considering the results, the present study provides new insights into the angiogenic and antiproliferative effects of ozonated water in a preclinical model of mammary carcinoma. Although these findings contribute to a better understanding of the biological mechanisms underlying ozone-induced tumor modulation, relevant gaps remain regarding its applicability in antineoplastic therapeutic protocols, particularly with respect to translational extrapolation to human clinical practice. Therefore, the use of ozone should be interpreted with caution, and the development of new therapeutic strategies may consider its application as an adjuvant or complementary approach, but not as a stand-alone modality for the treatment of these neoplasms.

## 5. Conclusions

In experimental SET models in mice, ozonated water promoted reduction in tumor cell proliferation in the medium term, whereas treatment discontinuation may have contributed to increase in microvascular density in the long term, which may also be related to a delayed pro-angiogenic effect. Therefore, caution is warranted in the use of O_3_ in oncological therapy, since, depending on the treatment protocol, both antineoplastic effects (reduction in cellular proliferation) and tumor-promoting effects (stimulation of vascular proliferation) may be observed.

## Figures and Tables

**Table 1:** Distribution of P2 mice with SET across groups treated with ozonated water (G1–G2), 0.9% saline solution (G3), and negative control (GCN), according to the number of applications (1–2x) and euthanasia time points (24 h, five, and 30 days after SET growth peak).

G1 Group Ozonated Water at 5 ppm			G2 Group Ozonated Water at 8 ppm			G3 Group 0.9% Saline Solution			GCN (*n*)
Subgroups G1 AP/EU	PT (*n*)	IT (*n*)	Subgroups G2 AP/EU	PT (*n*)	IT (*n*)	Subgroups G3 AP/EU	PT (*n*)	IT (*n*)	-
M1/G1-1/24 h	3	3	M1/G2-1/24 h	3	3	M1/G3-1/24 h	3	3	3
M2/G1-1/5 days	3	3	M2/G2-1/5 days	3	3	M2/G3-1/5 days	3	3	3
M3/G1-1/30 days	3	3	M3/G2-1/30 days	3	3	M3/G3-1/30 days	3	3	3
M4/G1-2/5 days	3	3	M4/G2-2/5 days	3	3	M4/G3-2/5 days	3	3	
M5/G1-2/30 days	3	3	M5/G2-2/30 days	3	3	M5/G3-2/30 days	3	3	-

G1: Groups treated with ozonated water at a concentration of 5 ppm; G2: Groups treated with ozonated water at a concentration of 8 ppm; G3: Vehicle control groups treated with 0.9% saline solution; GCN: Negative control groups; AP/EU: Number of applications/euthanasia time point; PT: Subcutaneous peritumoral administration route; IT: Intratumoral administration route.

**Table 2:** Correlation analysis between the variables microvascular density (anti-CD31) and cell proliferation (anti-Ki-67) in solid Ehrlich tumors in mice treated with ozonated water at different doses, numbers of applications, routes of administration, and evaluation points.

Evaluations	Correlation	*p*-Value
anti-CD31—IT × SCP	0.3007	<0.01
anti-CD31 (IT) × anti-Ki67	−0.0385	0.7048
anti-CD31 (SCP) × anti-Ki67	−0.0502	0.6215

Legend: CD31—IT × SCP: Correlation analysis between CD31 immunolabeling in intratumoral and peritumoral regions; CD31 (IT) × Ki-67: Correlation analysis between CD31 immunolabeling in intratumoral regions and Ki-67 immunolabeling; CD31 (SCP) × Ki-67: Correlation analysis between CD31 immunolabeling in peritumoral regions and Ki-67 immunolabeling.

## Data Availability

The original contributions presented in this study are included in the article. Further inquiries can be directed to the corresponding author.

## Abbreviations

The following abbreviations are used in this manuscript:

ET	Ehrlich Tumor
GCN	Negative Control Group
IT	Intratumoral
MVD	Microvessel Density
PT	Peritumoral
SCP	Peritumoral Subcutaneous
SET	Solid Ehrlich Tumor
